# In memory of Professor Iain Wilkinson: cognitive and neuroimaging endophenotypes in a consanguineous schizophrenia multiplex family

**DOI:** 10.1017/S0033291721005250

**Published:** 2023-05

**Authors:** Iain D. Wilkinson, Tariq Mahmood, Sophia Faye Yasmin, Anneka Tomlinson, Jamshid Nazari, Hamid Alhaj, Soumaya Nasser el din, Joanna Neill, Chhaya Pandit, Shahzad Ashraf, Alastair G. Cardno, Steven J. Clapcote, Chris F. Inglehearn, Peter W. Woodruff

**Affiliations:** 1 Academic Unit of Radiology, School of Medicine, University of Sheffield, Sheffield, UK; 2 Leeds & York Partnership NHS Foundation Trust, Leeds, UK; 3 Department of Psychiatry, University of Oxford, Oxford, UK; 4 South West Yorkshire NHS Foundation Trust, Wakefield, UK; 5 University of Sharjah, UAE; 6 Department of Neuroscience, School of Medicine, University of Sheffield, Sheffield, UK; 7 Division of Pharmacy and Optometry, University of Manchester, Manchester, UK; 8 Psychological & Social Medicine, Leeds Institute of Health Sciences, University of Leeds, Leeds, UK; 9 School of Biomedical Sciences, University of Leeds, Leeds, UK; 10 Division of Molecular Medicine, Leeds Institute of Medical Research, University of Leeds, Leeds, UK

**Keywords:** Cognition, connectivity, consanguinity, endophenotypes, fractional anisotropy, schizophrenia

## Abstract

**Background:**

Schizophrenia endophenotypes may help elucidate functional effects of genetic risk variants in multiply affected consanguineous families that segregate recessive risk alleles of large effect size. We studied the association between a schizophrenia risk locus involving a 6.1Mb homozygous region on chromosome 13q22–31 in a consanguineous multiplex family and cognitive functioning, haemodynamic response and white matter integrity using neuroimaging.

**Methods:**

We performed CANTAB neuropsychological testing on four affected family members (all homozygous for the risk locus), ten unaffected family members (seven homozygous and three heterozygous) and ten healthy volunteers, and tested neuronal responses on fMRI during an n-back working memory task, and white matter integrity on diffusion tensor imaging (DTI) on four affected and six unaffected family members (four homozygous and two heterozygous) and three healthy volunteers. For cognitive comparisons we used a linear mixed model (Kruskal–Wallis) test, followed by posthoc Dunn's pairwise tests with a Bonferroni adjustment. For fMRI analysis, we counted voxels exceeding the *p* < 0.05 corrected threshold. DTI analysis was observational.

**Results:**

Family members with schizophrenia and unaffected family members homozygous for the risk haplotype showed attention (*p* < 0.01) and working memory deficits (*p* < 0.01) compared with healthy controls; a neural activation laterality bias towards the right prefrontal cortex (voxels reaching *p* < 0.05, corrected) and observed lower fractional anisotropy in the anterior cingulate cortex and left dorsolateral prefrontal cortex.

**Conclusions:**

In this family, homozygosity at the 13q risk locus was associated with impaired cognition, white matter integrity, and altered laterality of neural activation.

## Introduction

The multifactorial aetiology of schizophrenia includes a substantial genetic component (Kahn et al., 2015), with a heritability of 60–80% (Cardno & Gottesman, 2000; Hilker et al., 2018; Sullivan, Kendler, & Neale, 2003). Considerable progress has been made in identifying common and rare risk loci in general population samples predominantly of European ancestry (Marshall et al., 2017; Ripke, Walters, & O'Donovan, 2020; Singh, Neale, & Daly, 2020). Recessively inherited risk alleles may be difficult to detect in general population samples, but are likely to be enriched in consanguineous multiplex families from endogamous populations (Mahmood et al., 2021). We previously identified a risk locus in such a family, involving a 6.1 Mb region of homozygosity on chromosome 13q22–31 (Mahmood et al., 2021).

Insights into pathophysiological processes stemming from risk alleles may be gained by investigating endophenotypes, including those based on cognitive functioning and neuroimaging parameters (Braff, Freedman, Schork, & Gottesman, 2007; Gottesman & Gould, 2003). For example, patients with schizophrenia show a broad range of cognitive function deficits (McCleery & Nuechterlein, 2019), including working memory deficits in patients (Forbes, Carrick, McIntosh, & Lawrie, 2009; Zanelli et al., 2019) and their unaffected siblings (Egan et al., 2001; Horan et al., 2008).

Impaired working memory can be assessed by n-back and spatial working memory tasks (Cannon et al., 2000; Glahn et al., 2003; Park, Holzman, & Goldman-Rakic, 1995). Functional magnetic resonance imaging (fMRI) has shown that haemodynamic response to neuronal activity, whilst undertaking working memory tasks, is reduced in the dorsolateral prefrontal cortex (DLPFC) and anterior cingulate cortex (ACC) in schizophrenia patients (Glahn et al., 2005). This quantitative difference in activation is seen as an indicator of the qualitative change in information processing within the DLPFC, which is not evident in manifest behaviour. Neural activity in these regions is also reduced in their unaffected relatives (Callicott et al., 2003) even when they have normal scores on tests of cognitive functioning (Thermenos et al., 2013). In addition, there is increasing interest in how neural markers of the DLPFC inefficiency and its connectivity observed during performance of working memory tasks in patients and their healthy relatives can be used as a putative endophenotype of schizophrenia (Deserno, Sterzer, Wustenberg, Heinz, & Schlagenhauf, 2012; Li et al., 2017; Potkin et al., 2009).

Since the early CT scanning studies, and subsequent structural MRI studies, there is now a large amount of evidence to support the presence of structural deficits in patients with schizophrenia (see Iliuta, Manea, Budisteanu, Ciobanu, and Manea, 2021 for a recent review). Most reports focus on grey matter deficits in the cerebral cortex including the hippocampus, which supports memory and higher cognitive function. In order to further explore the neural phenotype in this pedigree, we were additionally interested in applying DTI as a complementary measure of brain structure. Fractional anisotropy (FA) is the measurement of the direction water moving in and mean diffusion (MD) is the rate at which it moves. White matter regions have a higher rate of anisotropy but when fibre integrity is weakened the diffusion rate is faster and becomes more isotropic, which is thought to be due to damaged myelin sheaths and fibre membranes. These usually create a barrier, ensuring a steady diffusion speed (Ardekani et al., 2011). Research has suggested that patients with schizophrenia have disrupted white matter tracts and show faster MD speeds and lower FA, demonstrating that damage is not only structural in the white and grey matter but is also a problem in relation to brain connectivity. FA, therefore, is a way of measuring the integrity of brain circuits. A number of studies have demonstrated that people with schizophrenia have disrupted white matter tracts and have lower FA scores (Waters-Metenier & Toulopoulou, 2011). One of the affected areas is the cingulum bundle, particularly in the anterior area, where decreased FA could be the cause of decreased neuronal activity in the ACC in schizophrenia (Sun et al., 2003). These studies have given a valuable insight into the neural connectivity of schizophrenia and opened up a door for exploration by piecing together the results of previous and newer findings in different areas of schizophrenia research.

There is extensive literature that supports reversed laterality of brain function in schizophrenia, from handedness and neurocognitive tests, to activation studies on language and higher cognitive tasks (e.g. Sommer, Aleman, Ramsey, Bouma, and Kahn, 2018). Together with evidence of ‘hypofrontality’ in resting-state scans in patients with schizophrenia, and altered connectivity of DLPFC during the n-back task and recruitment of activity in the undamaged hemisphere in stroke patients, this supports the hypothesis that impaired function of one hemisphere may be compensated by the other in schizophrenia (Cramer, 2008; Potkin et al., 2009). We were therefore interested in exploring whether there could be altered laterality in brain activity on the n-back cognitive task, which is well known to activate the DLPFC.

As the prevailing diagnostic and classification systems DSM-5 and ICD10, which rely on clinical phenotype, are limited in their ability to ensure homogeneity of research samples, an endophenotype based system, namely Research Domain Criteria (RDoC) Framework, has been proposed to increase the homogeneity of study samples (Insel et al., 2010). Whilst the reliability and validity of RDoC is being established (Mahmood, 2020), Whalley et al. (2015) have found an innovative way of increasing the homogeneity of research samples by studying families with multiple cases of schizophrenia. Their multimodality approach combined molecular genetics, diffusion tensor imaging (DTI) and tractography to show aberrations of intra- and interhemispheric connectivity in patients with schizophrenia and their relatives. We have applied this approach to consanguineous families with multiple cases of schizophrenia from a community with a higher risk of psychosis (Saleem et al., 2019). In this paper, we report endophenotypic elucidation of a recessively behaving locus on 13q22−31, which was identified in a large extended family with multiple cases of schizophrenia (Mahmood et al., 2021). We investigated cognitive function, fMRI responses to a working memory task, and FA in this family.

**Fig. 1.**
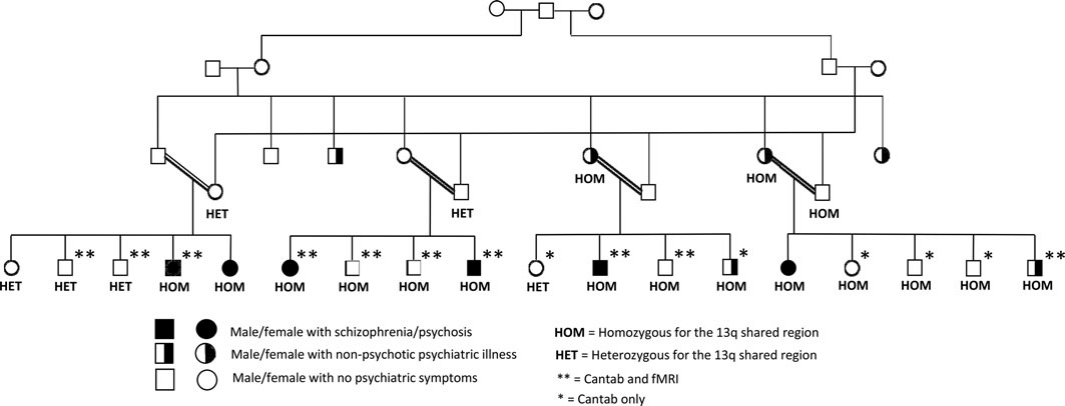
The multiplex consanguineous family discussed herein and described previously by Mahmood et al. (2021), with multiple cases of schizophrenia and non-psychotic psychiatric illness.

Members of the family (Fig. 1), consisting of four nuclear families formed by intermarriages among two sets of half cousins, took part in our studies. The family is from an immigrant community in which cousin marriages are common and the practice has been accentuated by their cultural insularity. Clinical research assessment was based on all available information including Schedules for Clinical Assessment in Neuropsychiatry (SCAN) (Wing et al., 1990) or Positive and Negative Syndrome Scale (PANSS) (Kay, Fiszbein, & Opler, 1987) research interview focusing on lifetime symptoms and review of clinical case records. Consensus main-lifetime DSM-5 diagnoses (APA, 2013) were based on independent ratings by TM and AC, with any discrepancies resolved by further review and discussion. Five family members had schizophrenia and one had other psychotic disorders with a history of delusions and manic symptoms. The onset of psychotic symptomatology occurred in the late teens or early twenties. Initially, affected individuals with schizophrenia presented with auditory hallucinations, paranoid delusions, and formal thought disorder. Later, they developed negative symptoms and cognitive decline. Males showed exacerbation of symptoms after taking cannabis, but females did not take cannabis.

All six family members with psychotic disorders were homozygous for a 6.1Mb region of homozygosity on chromosome 13q22–31, consistent with a recessively inherited risk allele (Mahmood et al., 2021). An additional 12 family members without a psychotic disorder were also homozygous for this region. On further psychiatric screening, two had DSM-5 major depressive disorder, one had anxiety symptoms including panic attacks, and one had a history of self-harm (Mahmood et al., 2021). The six other genotyped family members were heterozygous for the region and showed no notable mental health problems. Homozygosity for the region is likely to be rare in the general population, but seems to make a substantial contribution to the risk of psychotic disorder in this family (penetrance 33%). The region is bounded by SNPs rs17716584 (13q22.2) and rs7997648 (13q31.1) and contains protein-coding genes *KCTD12, ACOD1, CLN5, FBXL3, MYCBP2, SCEL, SLAIN1, EDNRB, POU4F1, RNF219, RBM26, NDFIP2* and *SPRY2*. There is no evidence of pathogenic coding or structural variants within the region. Based on RNA sequencing, six genes show differential expression. The leading candidate gene is *NDFIP2,* which influences T helper (Th) cell type 1 and Th2 phenotypes (Lund et al., 2007), which have been associated with schizophrenia (Schwarz, Chiang, Müller, & Ackenheil, 2001). Our findings suggest that a genetic variant in this region, which is likely to affect the function of one of the thirteen protein-coding genes, increases schizophrenia risk in those who are homozygous for it. However, it has not yet been possible to identify that variant or determine its precise biological effect.

**ETHICAL** approval for studies reported in this paper was granted by Yorkshire & Humber Regional Ethics Committee (13/YH/049).

## Material and methods

### Neurocognitive studies

The four patients (three males, one female) all had DSM-5 schizophrenia; had an age-at-onset between 17 and 21 years of age; illness durations between 7 and 26 years; PANSS total scores between 32 and 53; and were treated with: Clozapine 150 mg/day; Clozapine 300 mg/day; Aripiprazole 25 mg /day; Clozapine 250 mg/day and Aripiprazole 15 mg/day. Among the homozygous siblings without psychotic disorders, one had anxiety symptoms and one had a history of self-harm. To our knowledge, neither were treated with psychotropic medication. Patients (*n* = 4) and their unaffected siblings (unaffected homozygotes *n* = 7 and unaffected heterozygotes *n* = 3) underwent cognitive testing with the Cambridge Neuropsychological Test Automated Battery (CANTAB) (Strauss, Sherman, & Spreen, 2006) and were compared with healthy volunteers (*n* = 10, male 6, female 4, mean age 25.9 years, s.d. 5.46).

Healthy controls were recruited from local sports teams, businesses and students and matched to participants by ethnicity and sex. Controls were excluded if they had a history of mental illness, drug or alcohol abuse, learning disability, severe head injuries or other neurological illness. They were screened using the Mini-Mental State Exam (MMSE) (Cockrell & Folstein, 1998) and excluded if scoring <24. An interpreter was used to obtain consent and provide instructions for CANTAB tasks when required. A researcher (AT) trained in standardised administration and scoring of the battery administered and analysed all tasks. The researcher and interpreter were blinded to the genetic status of participants. These tests are language-independent, culturally neutral, require no prior technical knowledge, and have been standardised on large populations. Tests found to be sensitive to neurocognitive deficits in schizophrenia were selected from the CANTAB battery (Saleem et al., 2013). These included two tests assessing working memory – Spatial Recognition Memory (SRM) and Pattern Recognition Memory (PRM); a test assessing executive function – Intra-Extra Dimensional Shift (IED); a test assessing attention – Choice Reaction Time (CRT); and the Stockings of Cambridge (SOC) test assessing working memory and spatial planning. Tasks were completed on a touch-sensitive screen, taking approximately 45 min to complete, and each participant followed the same order. Scores of family members were compared to healthy controls. As these data did not meet the assumption of normality and because of the small sample size, a linear mixed model (Kruskal–Wallis) test was used for data analysis, followed by Dunn's pairwise tests. The Bonferroni adjustment was used as multiple tests were carried out. All analysis was conducted in SPSS (version 13).

### Neuroimaging

Functional and structural MRI images were acquired (four patients, four homozygous unaffected, two heterozygous unaffected, three healthy volunteers) during an n-back test conducted inside an MRI scanner (Ingenia 3.0 T, Phillips Healthcare, Best, Netherlands) with an MR compatible in-room LCD and fibre optic response button. We used a single shot epi sequence, total sequence time = 5 min 44s (140 dynamics– dynamic scan time 2.4 s); repetition time TR 2390, slice thickness 4 mm; Field of View 230 × 230, flip angle 90 degrees. The anatomical imaging consisted of axial T2W imaging (Turbo spin Echo, slice thickness 4 mm with on gap, turbo spin-echo factor 15, flip angle 90, reconstruction voxel size 0.53 mm × 0.53 mm, representative TR:TE = 3000:80 ms) and T1W volume imaging acquired in the coronal plane (slice thickness 1 mm, reconstructed voxel size 0.94 mm × 0.94 mm × 0.94 mm, representative TR:TE 8.2:3.8 ms).

An n-back test was used for functional imaging data during a working memory task. The n-back task comprised a presentation of a sequence of visual stimuli where the participant was required to indicate when the current stimulus matched one from *n* steps prior in the sequence. The factor *n* was adjusted from 1 to 3 to make the test less or more difficult respectively. Stimuli were presented in bold Georgia font, white text on a black background with 2 runs: 6 blocks in each run. The first run was 1-back *v.* 2-back and the second run was 1-back *v.* 3-back. Each block consisted of an instruction screen and 21 task trials which consisted of a single letter displayed for 1.9 s and a blank screen for 0.5 s, alternating. Each run lasted no more than 6 min, and just under 12 min in total. There were 2 practice runs which were identical to the 2 runs, but they only contained 4 blocks with 12 trials so that a baseline could be determined and the patient could conduct each run.

The boxcar-designed n-back task involved a presentation of a sequence of visual stimuli via an MR compatible in-room LCD screen, with the subject required to indicate, using a fibre-optic response button, when the current stimulus matched the one from *n* steps earlier in the displayed sequence. The load factor *n* was adjusted to make the task more or less difficult. Standard T1 weighted images were acquired to exclude the presence of incidental pathology. Resting-state and diffusion tensor images were obtained in the same session at rest.

We recorded the performance of each participant on a laptop, and included data from those who completed the task. However, the participants who were unable to complete the task stopped pressing the button before the end of the task.

**Diffusion Tensor Images** were run through FSL for DTI, and MATLAB and SPM 12 for pre-processing of fMRI. Configuration information for DTI data was extracted from enhanced DTI files, using an average of 32 gradient directions, a b-value of 800 and b0 images of 1. DTI data was run through Nordic Ice4 (Nordic Neurolabs) to determine FA and Mean Diffusivity (MD). Motion correction, eddy current correction and smoothing were applied prior to processing the data. A region of interest (ROI) analysis was run over three axial slices (which were then averaged), using neuroanatomical knowledge to determine the ACC and DLPFC areas in each individual participant, taking into account neuroanatomical differences. The results from ROI analysis were analysed with SPSS to average and acquire standard deviations for the FA and MD values.

## Results

### Neurocognitive studies

Patients were regularly assessed clinically by a psychiatrist in a clinic or ward (TM). Initially, affected individuals with schizophrenia presented with prominent auditory hallucinations, paranoid delusions, and formal thought disorder. Later, negative symptoms and cognitive decline were also prominent.

**Fig. 2.**
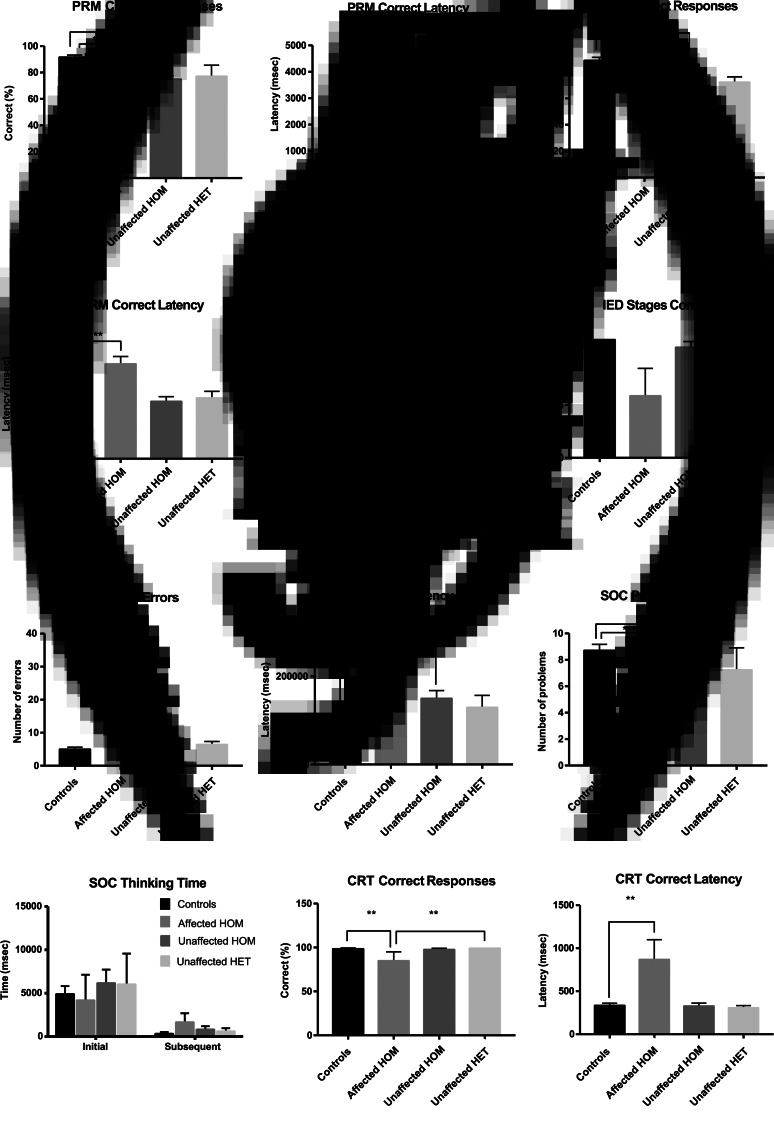
Cognitive (CANTAB) testing. Results are expressed as the mean ± s.e.m.. Asterisks (***p < 0.001; **p < 0.01; *p < 0.05) indicate significant differences between groups. (*a*) In the PRM test, both affected and unaffected homozygotes made significantly fewer correct responses (%) than controls (*p* < 0.001, *p* < 0.001). (*b*) Affected homozygotes also took significantly longer than controls to choose the correct pattern (correct latency in msec) (*p* < 0.001). (*c*) In the SRM test, both affected and unaffected homozygotes made significantly fewer correct responses (%) than controls (*p* < 0.01, *p* < 0.01). (*d*) Affected homozygotes also took significantly longer to choose the correct pattern than controls (correct latency in msec) (*p* < 0.01). (*e*) In the IED test, affected homozygotes made significantly more errors in total (*p* < 0.01). (*f*) No significant differences between the groups were observed for stages completed. (*g*) No significant differences between the groups were observed for pre-ED errors. (*h*) Affected homozygotes (*p* < 0.01) and unaffected homozygotes (*p* < 0.01) also took significantly longer to make a response compared with controls (correct latency, msec). (*i*) In the SOC test, affected and unaffected homozygotes solved fewer problems in the minimum number of moves than controls (*p* < 0.001, *p* < 0.01), but (j) no significant differences in thinking time (msec) were observed between groups. (*k*) In the CRT, affected homozygotes made significantly fewer correct responses (%) than heterozygotes (*p* < 0.01) and controls (*p* < 0.01). (*l*) Affected homozygotes took longer to make a correct response (correct latency in msec) than controls (*p* < 0.01). PRM, Pattern recognition memory; SRM, Spatial Recognition Memory; IED, Intra-extra Dimensional Shift; SOC, Stockings of Cambridge test; CRT, Choice Reaction Time test.

In all five cognitive tests, affected homozygotes scored significantly lower for cognitive ability than controls (Fig. 2). This could in part be a consequence of antipsychotic medication. However, in four of the five tests (PRM, SWM, IED and SOC), unaffected homozygotes, who are not on antipsychotic medication, also scored significantly lower than controls. Scores for affected and unaffected homozygotes did not differ significantly in any cognitive tests. Scores for heterozygotes did not differ significantly from controls or from unaffected homozygotes in any cognitive test, and only differed from unaffected homozygotes in one test (CRT Correct Responses), but this may reflect the low number of heterozygotes tested (*n* = 3). Further details of these results are given in Supplementary Tables 1–4.

### Neuroimaging

#### Functional magnetic imaging

**Fig. 3.**
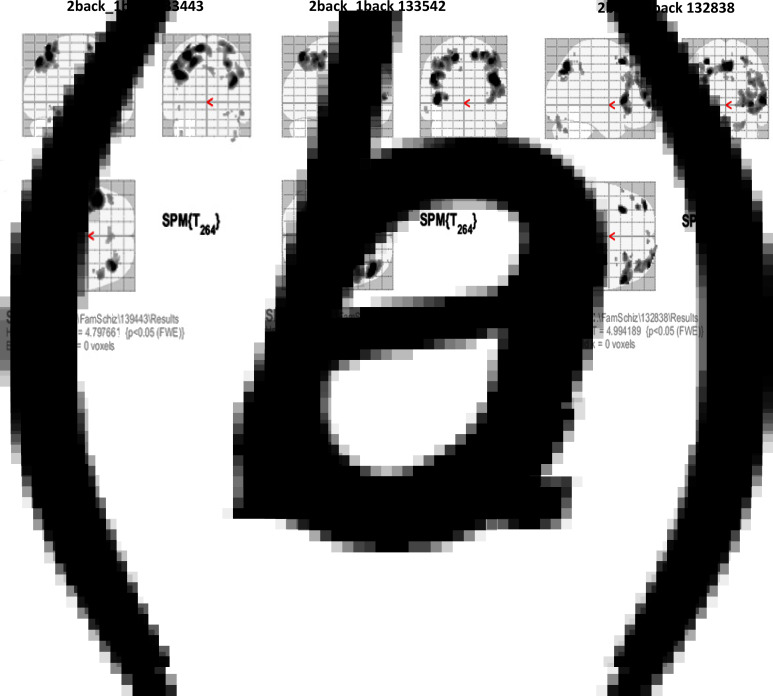
fMRI testing during a n-back working memory task. Examples are shown of fMRI BOLD activation in: (*a*), a healthy volunteer; (*b*), a heterozygote; and (*c*), an unaffected homozygote. The SPM maps shown are *z* maps of statistical differences between conditions within the brain. Prefrontal activation of voxel clusters >100 shows left>right activation bias (1028 *v.* 449) in a healthy volunteer and left<right in heterozygous (771 *v.* 1387) and homozygous relatives (0 *v.* 1032).

No obvious anatomical differences were observed between homozygotes and controls on MRI brain scans. fMRI analysis revealed pre-frontal cortical activation predominantly on the left in healthy controls, with a bias towards right activation in unaffected heterozygous and homozygous family members. Affected homozygotes had difficulty with the task and activation of the prefrontal cortex was minimal (Fig. 3 and Table 1).

**Table 1.** Left and right prefrontal activation levels measured by fMRI at 3T (Ingenia 3.0T) whilst healthy volunteers, unaffected heterozygotes and unaffected homozygotes performed the *n*-back working memory tasks

Subject	Test	Left activation	Right activation
Control 1	2 back	2039	1278
	3 back	*	*
Control 2	2 back	1028	449
	3 back	*	*
Control 3	2 back	*	*
	3 back	745	202
Heterozygote 1	2 back	771	1387
	3 back	847	1506
Heterozygote 2	2 back	667	282
Homozygote 1	2 back	0	1032
	3 back	*	*
Homozygote 2	2 back	*	*
	3 back	46	96
Homozygote 3	2 back	*	*
	3 back	1641	1682

For SPM brain maps set at threshold of *p* < 0.05 (corrected) to allow for multiple comparisons, values are total voxels in prefrontal cortex where clusters were >100 voxels in any region. For maps where no clusters exceeded 100 voxels, the total count of all voxels is included. Affected homozygotes had difficulty with the task and activation of prefrontal cortex was minimal, so their results are not included. Not all tests reached required quality thresholds in all subjects, and only results of those which did are given. Heterozygote 2 showed a left > right pattern overall (667 *v.* 282), but the right > left pattern remained for the superior prefrontal region (282 *v.* 0) on the 2-back task.

#### Diffusion tensor imaging

FA showed a consistent downwards gradient in the anterior cingulate cortex (Table 2). The patients had the lowest scaler score (an average of 0.087), homozygous unaffected relatives scored a little higher (an average of 0.159), heterozygous unaffected relatives were higher still (an average of 0.177), and healthy controls had the highest FA score (an average of 0.195) (Table 2). A similar but less consistent trend was seen in the left dorsolateral prefrontal cortex (DLPFC-L) and right dorsolateral prefrontal cortex (DLPFC-R) (Table 2). Mean diffusivity scores were higher for the patients and lower for the healthy volunteers (Table 3).

**Table 2.** Fractional anisotropy in ACC, DLPFC-L and DLPFC-R shows downward trend from healthy volunteers to heterozygous relatives, homozygous unaffected relatives and patients

	ACC Mean (s.d.)	DLPFC-R	DLPFC-L
Healthy controls	0.195 (0.267)	0.142 (0.061)	0.161 (0.066)
Heterozygous	0.177 (0.155)	0.140 (0.037)	0.112 (0.030)
Homozygous	0.159 (0.114)	0.139 (0.044)	0.137 (0.054)
Patient	0.087 (0.028)	0.123 (0.074)	0.121 (0.056)

ACC, anterior cingulate cortex; DLPFC-R, dorsolateral prefrontal cortex right; DLPFC-L, dorsolateral prefrontal cortex left.

**Table 3.** Mean Diffusivity in ACC, DLPFC-L and DLPFC-R shows upward trend from healthy controls to heterozygous relatives, homozygous unaffected relatives and patients

	ACC Mean (s.d.)	DLPFC – R	DLPFC – L
Healthy controls	97.869 (37.057)	90.693 (15.162)	89.308 (8.533)
Homozygous	99.800 (36.954)	88.540 (14.500)	82.662 (27.373)
Heterozygous	107.630 (15.696)	92.893 (9.633)	92.478 (19.736)
Patient	121.762 (15.665)	100.424 (18.892)	92.501 (16.082)

ACC, anterior cingulate cortex; DLPFC-R dorsolateral prefrontal cortex right; DLPFC-L, dorsolateral prefrontal cortex left.

## Discussion

We report analysis of cognitive and neuroimaging parameters in an extended consanguineous family with multiple cases of schizophrenia. There is evidence that schizophrenia in this family is at least in part caused by a recessively inherited, high penetrance susceptibility allele or haplotype on chromosome 13q22–31 (Mahmood et al., 2021). Multiple lines of evidence suggest an endophenotype of reduced cognitive function in homozygotes, both those with and those without a psychotic disorder. Other mental health problems were documented in four of the twelve apparently unaffected homozygotes. On CANTAB testing both unaffected and affected homozygotes performed significantly worse than controls. fMRI suggested a bias in activation, with activation shifting from the left towards the right side of the prefrontal cortex in both homozygous and heterozygous haplotype carriers *v.* controls during a working memory task, with the greatest shift in homozygotes and an intermediate level in heterozygotes. Reversed brain laterality in schizophrenia is well documented in language and cognitive regions including DLPFC (Gur & Chin, 1999; Hirnstein & Hugdahl, 2014; Sommer et al., 2018), which may be considered a compensatory mechanism for deficient left-sided function (Minzenberg, ALaird, Thelen, Carter, & Glahn, 2009) or altered biochemical basis for synaptic function on the right (Psomiades et al., 2018). Cognitive impairment has also been documented in close relatives of schizophrenia patients (Egan et al., 2001). However, the modest numbers in each genotype category limit the significance of our data, particularly for fMRI, so these findings should be considered preliminary. Nevertheless, these observations point to a spectrum of severity in carriers for the risk haplotype. Homozygotes exhibited phenotypes ranging from lowered cognitive function in the absence of mental health problems (*n* = 8), through milder mental health problems (*n* = 4) to the most severely affected individuals who have psychosis (*n* = 6). This may indicate that the 13q risk haplotype acts in concert with environmental and other genetic factors, with cannabis use a possible contributory factor in males (Di Forti et al., 2019; Mahmood et al., 2021). It should be noted that cannabis use could potentially also influence cognitive function. We are not aware of other environmental risk factors in family members but these cannot be excluded. Although preliminary, our findings are in line with ongoing work that reports aberrant brain connectivity in schizophrenia (Fornito, Zaleski, Pantelis, & Bullmore, 2012) and links between brain connectivity measures and cognitive function in patients with schizophrenia and their relatives (Li et al., 2017).

Our findings are consistent with earlier reports of aberrant white matter connectivity in cases of schizophrenia, particularly in ACC and left DLPFC (Newman & McGaughy, 2011), brain areas which underpin attention (Newman, Creer, & McGaughy, 2015) and working memory (Barbey, Koenigs, & Grafman, 2013). The fact that similar changes are seen in unaffected homozygous family members discounts the possibility of these being the effects of psychotic illness or antipsychotic treatment (Lett, Voineskos, Kennedy, Levine, & Daskalakis, 2014).

This methodology, which utilises a large consanguineous family with multiple affected individuals, has its strengths in that it increases the genetic and phenotypic homogeneity compared with general population samples, thus the power to detect deficits in working memory, neural activation and connectivity are enhanced (Gottesman & Gould, 2003). There are some inherent disadvantages, most notable being the small size of study samples, which is unavoidable when faced with the challenge of recruiting within a family. Additionally, the underlying risk allele is likely to be rare in the general population, which may limit generalisability, although there is evidence of some overlap in genes showing common and rare genetic associations in schizophrenia (Ripke et al., 2020; Singh et al., 2020). Although our preliminary findings may be limited to consanguineous families, these families are not uncommon in the community studied. Given the highly complex, polygenic nature of genetic risk in schizophrenia and other forms of psychiatric illness, our findings may not prove widely generalisable to others with these conditions. However, a similar approach may be used to dissect out loci in similar families.

We used the n-back task as a tried and tested cognitive test of working memory. However, we recognise that there are limitations to drawing conclusions as to its exact cognitive mechanism, as is illustrated in a recent debate about the extent to which it relies upon similar neural processes in different people (Eich, Nee, Insel, Malapani, & Smith, 2014). Also, we did not match for educational level, although participants were drawn from similar family backgrounds. However, our preliminary findings may add some weight to the ongoing work looking for links between genes and the brain substrates for cognitive dysfunction in schizophrenia (Petralia et al., 2020).

## Conclusions

To our knowledge, this is the first study which looks into neurocognitive and neuroimaging endophenotypes in a consanguineous family with multiple cases of schizophrenia. It shows the degree of genetic risk, i.e. homozygosity for the 13 q22–31 locus, to influence the cognitive functions, neural activation and white matter integrity in this family and supports our premise that the study of multiply affected families from a community with a higher risk of psychosis can assist in finding genes of major effect and elucidating their correlations with schizophrenia endophenotypes.

## Supplementary material

For supplementary material accompanying this paper visit https://doi.org/10.1017/S0033291721005250. click here to view supplementary material

## References

[ref1] American Psychiatric Association. (2013). Diagnostic and statistical manual of mental disorders (5th ed.). Arlington: VA: American Psychiatric Association. 10.1176/appi.books.9780890425596

[ref2] Ardekani, B. A., Tabesh, A., Sevy, S., Robinson, D. G., Bilder, R. M., & Szeszko, P. R. (2011). Diffusion tensor imaging reliably differentiates patients with schizophrenia from healthy volunteers. Human Brain Mapping, 32(1), 1–9. doi:10.1002/hbm.20995 20205252 PMC2896986

[ref3] Barbey, A. K., Koenigs, M., & Grafman, J. (2013). Dorsolateral prefrontal contributions to human working memory. Cortex, 49, 1195–1205. doi:10.1016/j.cortex.2012.05.022 22789779 PMC3495093

[ref4] Braff, D. L., Freedman, R., Schork, N.J., & Gottesman, I. I. (2007). Deconstructing schizophrenia: An overview of the use of endophenotypes in order to understand a complex disorder. Schizophrenia Bulletin, 33, 21–32. doi:10.1093/schbul/sbl049 17088422 PMC2632293

[ref5] Callicott, J. H., Egan, M. F., Mattay, V. S., Bertolino A., Bone, A. D., Verchinksi, B., & Weinberger, D. R. (2003). Abnormal fMRI response of the dorsolateral prefrontal cortex in cognitively intact siblings of patients with schizophrenia. American Journal of Psychiatry, 160, 709–719. doi:10.1176/appi.ajp.160.4.709 12668360

[ref6] Cannon, T. D., Huttunen, M. O., Lonnqvist, J., Tuulio-Henriksson, A., Pirkola, T., Glahn, D., … Koskenvuo, M. (2000). The inheritance of neuropsychological dysfunction in twins discordant for schizophrenia. American Journal of Human Genetics, 67(2), 369–382. doi:10.1086/303006 10880296 PMC1287184

[ref7] Cardno, A. G., & Gottesman, I. I. (2000). Twin studies of schizophrenia: From bow-and-arrow concordances to Star Wars Mx and functional genomics. American Journal of Medical Genetics, 97(1), 12–17. Retrieved from https://pubmed.ncbi.nlm.nih.gov/10813800. 10813800

[ref8] Cockrell, J. R., & Folstein, M. F. (1998). Mini-mental state examination (MMSE). Psychopharmacological Bulletin, 24, 689–692. Retrieved from https://pubmed.ncbi.nlm.nih.gov/3249771/. 3249771

[ref9] Cramer, S. C. (2008). Repairing the human brain after stroke: I. Mechanisms of spontaneous recovery. Annals of Neurology, 63, 3. doi:10.1002/ana.21393 18383072

[ref10] Deserno, L., Sterzer, P., Wustenberg, T., Heinz, A., & Schlagenhauf, F. (2012). Reduced prefrontal-parietal effective connectivity and working memory deficits in schizophrenia. The Journal of Neuroscience, 32(1), 12–20. doi:10.1523/jneurosci.3405-11.2012 22219266 PMC6621317

[ref11] Di Forti, M., Quattrone, D., Freeman, T.P., Tripoli, G., Gayer-Anderson, C., & Quigley, H., … the EU-GEI WP2 Group. (2019). The contribution of cannabis use to variation in the incidence of psychotic disorder across Europe (EU-GEI): A multicentre case-control study. The Lancet, 6(5), 427–436. doi:10.1016/S2215-0366(19)30048-3 30902669 PMC7646282

[ref12] Egan, M., Goldberg, T., Gscheidle, T., Weirich, M., Rawlings, R., Hyde, T. M., … Weinberger, D. R. (2001). Relative risk for cognitive impairments in siblings of patients with schizophrenia. Biological Psychiatry, 50(2), 98–107. doi:10.1016/s0006-3223(01)01133-7 11527000

[ref13] Eich, T. S., Nee, D. E., Insel, C., Malapani, C., & Smith, E. E. (2014). Neural correlates of impaired cognitive control over working memory in schizophrenia. Biological Psychiatry, 76(2), 146–153. doi:10.1016/j.biopsych.2013.09.032 24239131 PMC4984253

[ref14] Forbes, N. F, Carrick, L.A., McIntosh, A. M., & Lawrie, S. M. (2009). Working memory in schizophrenia: A meta-analysis. Psychological Medicine, 39, 889–905. doi:0.1017/S0033291708004558 18945379 10.1017/S0033291708004558

[ref15] Fornito, A., Zaleski, A., Pantelis, C., & Bullmore, E. (2012). Schizophrenia, neuroimaging and connectomics. Neuroimage, 62(4), 296–314. doi:10.1016/j.neuroimage.20112011.12.090 22387165

[ref16] Glahn, D., Ragland, J., Abramoff, A., Barrett, J., Laird, A. R., Beardon, C., & Velligan, D. (2005). Beyond hypofrontality: A quantitative meta-analysis of functional neuroimaging studies of working memory in schizophrenia. Journal of Human Brain Mapping, 25, 60–69. doi:10.1002/hbm.20138 15846819 PMC6871703

[ref17] Glahn, D. C., Therman, S., Manninen, M., Huttunen, M., Kaprio, J., Lönnqvist, J., & Cannon, T. D. (2003). Spatial working memory as an endophenotype for schizophrenia. Biological Psychiatry, 53, 62–626. doi:10.1016/s0006-3223(02)01641-4 12679242

[ref18] Gottesman, I., & Gould, T. D. (2003). The endophenotype concept in psychiatry: Etymology and strategic intentions. American Journal of Psychiatry, 160(4), 636–645. doi:10.1176/appi.ajp.160.4.636 12668349

[ref19] Gur, R. E., & Chin, S. (1999). Laterality in functional brain imaging studies of schizophrenia. Schizophrenia Bulletin, 25, 141–156. doi:10.1093/oxfordjournals.schbul.a033361 10098918

[ref20] Hilker, R., Helenius, D., Fagerlund, B., Skytthe, A., Christensen, K., Werge, T. M., … Glenthøj, B. (2018). Heritability of schizophrenia and schizophrenia spectrum based on the nationwide Danish twin register. Biological Psychiatry, 83(6), 492–498. doi:10.1016/j.biopsych.2017.08.017 28987712

[ref21] Hirnstein, M., & Hugdahl, K. (2014). Excess of non-right-handedness in schizophrenia: Meta-analysis of gender effects and potential biases in handedness assessment. British Journal of Psychiatry, 205(4), 260–267. doi:10.1192/bjp.bp.113.137349 25274314

[ref22] Horan, W. P., Braff, D. L., Nuechterlein, K. H., Sugar, C. A., Cadenhead, K. S., Calkins, M. E., … Green, M. F. (2008). Verbal working memory impairments in individuals with schizophrenia and their first-degree relatives: Findings from the consortium on the genetics of schizophrenia. Schizophrenia Research, 103(1–3), 218–228. doi:10.1016/j.schres.2008.02.014 18406578 PMC2529172

[ref23] Iliuta, F.P., Manea, M. C., Budisteanu, M., Ciobanu, A. M., & Manea, M. (2021). Magnetic resonance imaging in schizophrenia: Luxury or necessity? (Review) Experimental and Therapeutic Medicine, 22, 1. doi:10.3892/etm.2021.10197 PMC8145262 34055064

[ref24] Insel, T., Garvey, M., Heinssen, R., Pine, D. S, Quinn, K., Sainslow, C., & Wang, P. (2010). Research domain criteria (RDoC): Toward a new classification framework for research on mental disorders. Americal Journal of Psychiatry, 167, 748–751. doi:10.1176/appi.ajp.2010.09091379 20595427

[ref25] Kahn, R.S., Sommer, I. E., Murray, R. M., Meyer-Lindenberg, A., Weinberger, D.R., Cannon, T. D., … Thomas, & R. Insel (2015). Schizophrenia. Nature Reviews Disease Primers, 1, 15067. Retrieved from https://www.nature.com/articles/nrdp201567. 10.1038/nrdp.2015.67 27189524

[ref26] Kay, S. R., & Fiszbein, A., & Opler, L. A. (1987). The positive and negative syndrome scale (PANSS) for schizophrenia. Schizophrenia Bulletin, 13(2), 261–276. doi:10.1093/schbul/13.2.261 3616518

[ref27] Lett, T. A., Voineskos, A. N., Kennedy, J. L., Levine, B., & Daskalakis, Z. J. (2014). Treating working memory deficits in schizophrenia: A review of the neurobiology. Biological Psychiatry, 75(5), 361–370. doi:10.1016/j.biopsych.2013.07.026 24011822

[ref28] Li, P., Fan, T-T., Zhao, R-J., Han, Y., Shi, L., Sun, H-Q., … Lu, L. (2017) Altered brain network connectivity as a potential endophenotype of schizophrenia. Nature Scientific Reports, 7, 5483. doi:10.1038/s41598-017-05774-3 PMC5511161 28710394

[ref29] Lund, R. J., Löytömäki, M., Naumanen, T., Dixon, C., Chen, Z., Ahlfors, H., … Lahesmaa, R. (2007). Genome-wide identification of novel genes involved in early Th1 and Th2 cell differentiation. Journal of Immunology *(*Baltimore, Md*., 1950)*, 178(6), 3648–3660. Retrieved from 10.4049/jimmunol.178.6.3648. 17339462

[ref30] Mahmood, T. (2020). Biomarkers in psychiatry. British Medical Bulletin, 135(1), 23–27. doi:10.1093/bmb/Idaa019 32676652

[ref31] Mahmood, T., El-Asrag, M. E., Poulter, J. A., Cardno, A. G., Tomlinson, A., Ahmed, S., … Inglehearn, C. F. (2021). A recessively inherited risk locus on chromosome 13q22–31 conferring susceptibility to schizophrenia. Schizophrenia Bulletin, 47(3), 796–802. doi:10.1093/schbul/sbaa161 33159203 PMC8084434

[ref32] Marshall, C. R., Howrigan, D. P., Merico, D., Thiruvahindrapuram, B., Wu, W., & Greer, D. S., … CNV and Schizophrenia Working Groups of the Psychiatric Genomics Consortium. (2017). Contribution of copy number variants to schizophrenia from a genome-wide study of 41 321 subjects. Nature Genetics, 49(1), 27–35. doi:10.1038/ng.3725 27869829 PMC5737772

[ref33] McCleery, A., & Nuechterlein, K. H. (2019). Cognitive impairment in psychotic illness: Prevalence, profile of impairment, developmental course, and treatment considerations. Dialogues of Clinical Neuroscience, 21(3), 239–248. doi:10.31887/DCNS.2019.21.3/amccleery PMC6829172 31749648

[ref34] Minzenberg, M. J., ALaird, A. R., Thelen, S., Carter, C. S., & Glahn, D. C. (2009). Meta-analysis of 41 functional neuroimaging studies of executive function in schizophrenia. Archives of General Psychiatry, 66(8), 811–822. doi:10.1001/archgenpsychiatry.2009.91 19652121 PMC2888482

[ref35] Newman, L. A., Creer, D. J., & McGaughy, J. (2015). Cognitive control and the anterior cingulate cortex: How conflicting stimuli affect attentional control in the rat. Journal of Physiology *(*Paris*)*, 109(1–3), 95–103. doi:10.1016/j.jphysparis.2014.06.004 25051488 PMC4298471

[ref36] Newman, L. A., & McGaughy, J. (2011). Attentional effects of lesions to the anterior cingulate cortex: How prior reinforcement influences distractibility. Behavioural Neuroscience, 125(3), 360–371. doi:10.1037/a0023250 PMC3109123 21480690

[ref37] Park, S., Holzman, P. S, & Goldman-Rakic, P. (1995). Spatial working memory differences in relatives of schizophrenic patients. Archives of General Psychiatry, 52, 821–828. doi:10.1001/archpsyc.1995.03950220031007 7575101

[ref38] Petralia, M. C, Ciurleo, R., Saraceno, A., Pennisi, M., Basile, M S., Fagone, P., … Cavalli, E. (2020). Meta-Analysis of transcriptomic data of dorsolateral prefrontal cortex and of peripheral blood mononuclear cells identifies altered pathways in schizophrenia. Genes, 11(4), 390–406. doi:10.3390/genes11040390 32260267 PMC7230488

[ref39] Potkin, S. G., Turner, J. A., Brown, G. G., McCarthy, G., Greve, D. N., Glover, G. H., … Lim, K. O. (2009). Working memory and DLPFC inefficiency in schizophrenia: The FBIRN study. Schizophrenia Bulletin, 35(1), 19–31. doi:10.1093/schbul/sbn162 19042912 PMC2643959

[ref40] Psomiades, M., Mondino, M., Fonteneau, C., Bation, R., Haesebaert, F., Suaud-Chagny, M-F., & Brunelin, J. (2018). N-Acetyl-Aspartate in the dorsolateral prefrontal cortex in men with schizophrenia and auditory verbal hallucinations: A 1.5 T magnetic resonance spectroscopy study. Nature Scientific Reports, 8, 4133. doi:10.1038/s41598-018-22597-y PMC5841306 29515172

[ref41] Ripke, S., Walters, J., & O'Donovan, M. C. (2020). Mapping genomic loci prioritises genes and implicates synaptic biology in schizophrenia. doi:10.1101/2020.09.12.20192922

[ref42] Saleem, M., Brewin, A., Ding, C., Nazari, J., Garnham, M., Robinson, J., … Mahmood, T. (2019). Risk of psychosis in Yorkshire South Asians. Journal of Psychiatric Intensive Care, 15(2), 117–121. doi:10.20299/JPI.2019.007

[ref43] Saleem, M., Harte, M., Marshall, K., Scally, A., Brewin, A., & Neill, J. (2013). First episode psychosis patients show impaired cognitive function – a study of a South Asian population in the UK. Journal of Psychopharmacology, 27(4) 366–373. doi:10.1177/0269881113477746 23427189

[ref44] Schwarz, M. J., Chiang, S., Müller, N., & Ackenheil, M. (2001). T-helper-1 and T-helper-2 responses in psychiatric disorders. Brain Behavior and Immunity, 15(4), 340–370. doi:10.1006/brbi.2001.0647 11782103

[ref45] Singh, T., Neale, B. M., & Daly, M. J. on behalf of the Schizophrenia Exome Meta-Analysis (SCHEMA) Consortium. (2020). Exome sequencing identifies rare coding variants in 10 genes which confer substantial risk for schizophrenia. doi:10.1101/2020.09.18.20192815

[ref46] Sommer, I., Aleman, A., Ramsey, N., Bouma, A., & Kahn, R. (2018) Handedness, language lateralisation and anatomical asymmetry in schizophrenia: Meta-analysis. The British Journal of Psychiatry, 178(4), 344–351. doi:10.1192/bjp.178.4.344 11282814

[ref47] Strauss, E., Sherman, E.M. S., & Spreen, O. (2006). CANTAB: A compendium of neuropsychological tests: Administration, norms, and commentary (3rd ed., pp. 415–424). New York: Oxford University Press. doi:10.1080/09084280701280502

[ref48] Sullivan, P. F., Kendler, K. S., & Neale, M. C. (2003). Schizophrenia as a complex trait: Evidence from a meta-analysis of twin studies. Archives of General Psychiatry, 60(12), 1187–1192. doi:10.1001/archpsyc.60.12.1187 14662550

[ref49] Sun, Z., Wang, F., Cui, L., Breeze, J., Du, X., Wang, X., … Zhang, D. (2003). Abnormal anterior cingulum in patients with schizophrenia. Neuroreport, 14(14), 1833–1836. doi:10.1097/00001756-200310060-00015 14534430

[ref50] Thermenos, H. W., Keshavan, M., Juelich, R., Molokotos, E., Whitfield-Gabrielli, S., Brent, B., … Seidman, L. (2013). A review of neuroimaging studies of young relatives of individuals with schizophrenia: A developmental perspective from schizotaxia to schizophrenia. American Journal of Medical Genetics, Part B, 162B, 604–635. doi:10.1002/ajmg.b.32170 24132894

[ref51] Waters-Metenier, S. L., & Toulopoulou, T. (2011). Putative diffusion tensor imaging endophenotypes in schizophrenia: A review of the early evidence. Future Neurology, 6(3), 415–433. doi:10.2217/fnl.11.16

[ref52] Whalley, H. C., Dimitrova, R., Sprooten, E., Dauvermann, M. R., Romaniuk, L., Duff, B., … Lawrie, S. M. (2015). Effects of a balanced translocation between chromosomes 1 and 11 disrupting the DISC1 locus on white matter integrity. PLoS One, 10(6), e0130900. doi:10.1371/journal.pone.0130900 26102360 PMC4477898

[ref53] Wing, J. K., Babor, T., Brugha, T., Burke, J., Cooper, J. E., Giel, R., … Sartorius, N. (1990). SCAN. Schedules for clinical assessment in neuropsychiatry. Archives of General Psychiatry, 47(6), 589–593. doi:10.1001/archpsyc.1990.01810180 2190539

[ref54] Zanelli, J., Mollon, J., Sandin, S., Morgan, C., Dazzan, P., Pilecka, I., … Reichenberg, A. (2019). Cognitive change in schizophrenia and other psychoses in the decade following the first episode. American Journal of Psychiatry, 176(10), 811–819. doi:10.1176/appi.ajp.2019.18091088 31256609

