# Supplementary material for: In memory of Professor Iain Wilkinson: cognitive and neuroimaging endophenotypes in a consanguineous schizophrenia multiplex family
Source: Psychol Med. 2022 Feb 7;53(7):3178–86. doi: 10.1017/S0033291721005250 (PMC10235651; doi:10.1017/S0033291721005250)
Supplement: Supplementary file 1 [file S0033291721005250sup.zip › S0033291721005250sup001.docx]

|  | **Pattern Recognition Memory** | | **Spatial Recognition Memory** | |
| --- | --- | --- | --- | --- |
|  | % correct | Mean Correct latency | % Correct | Mean Correct latency |
| **Controls** | Mean 92.21  SD 5.64 | 1666.64  541.58 | 89.33  9.4 | 1554.99  778.2 |
| **Family unaffected heterozygotes** | Mean 67.7  SD 22.91  ES 1.71 | 2486.09  865.09  1.84 | 70  14.71  1.60 | 2180.66  436.44  1.03 |
| **Family**  **Unaffected homozygotes** | Mean 75.60  SD 5.6  ES 2.95 | 2222.43  1008.67  0.71 | 72.86  9.06  1.60 | 2200.64    396.24  1.09 |
| **Patients** | Mean 53.12  SD 8.59  ES 5.49 | 4237.52  792.4  3.85 | 55.0  15.81  2.72 | 3620.85  506.49  3.21 |

**Table 1 Pattern and Spatial Recognition Memory –** impairment is most marked in patients and less so in unaffected homozygotes and heterozygotes.

SD = Standard Deviation ES = Effect Size
