# Supplementary material for: In memory of Professor Iain Wilkinson: cognitive and neuroimaging endophenotypes in a consanguineous schizophrenia multiplex family
Source: Psychol Med. 2022 Feb 7;53(7):3178–86. doi: 10.1017/S0033291721005250 (PMC10235651; doi:10.1017/S0033291721005250)
Supplement: Supplementary file 1 [file S0033291721005250sup.zip › S0033291721005250sup002.docx]

**Table 2 Executive Function - Intra / Extra Dimensional Shift (IED)** – Compared with controls patients are slower and make more errors. Unaffected homozygotes and heterozygotes are impaired to a lesser degree.

| IED | **Total Errors**  **Adjusted** | **Stages Completed** | **Pre-ED Errors** | **Total Latency** |
| --- | --- | --- | --- | --- |
| **Controls** | Mean 12.60  SD 4.86 | 9.00  0.00 | 5.23  2.11 | 110287.93  153431.05 |
| **Family unaffected Heterozygotes** | Mean 39.5  SD 25.03  ES 1.79 | 8  1.15  1.73 | 6.25  1.25  0.60 | 141343.75  40754.22  1.29 |
| **Family**  **Unaffected HZ siblings** | Mean 27.71  SD 19.66  ES 1.23 | 8.43  0.97  1.17 | 7.0  4.5  0.53 | 151860.00  42056.12  0.42 |
| **Patients** | Mean 122.00  SD 95.53  ES 2.17 | 4.75  4.03  2.12 | 23.50  21.94  1.51 | 212868.25  66316.34  0.93 |

SD = Standard Deviation ES = Effect Size
