# Supplementary material for: In memory of Professor Iain Wilkinson: cognitive and neuroimaging endophenotypes in a consanguineous schizophrenia multiplex family
Source: Psychol Med. 2022 Feb 7;53(7):3178–86. doi: 10.1017/S0033291721005250 (PMC10235651; doi:10.1017/S0033291721005250)
Supplement: Supplementary file 1 [file S0033291721005250sup.zip › S0033291721005250sup003.docx]

**Table 3 Executive Function – Stockings of Cambridge (SOC) –** Patients spend less time thinking about a problem but need more moves and solve fewer problems.

|  | **Mean Initial Thinking Time (5 moves)** | **Mean moves (to solve a 5 move problem)** | **Problems solved in minimum moves** | **Mean subsequent thinking time** |
| --- | --- | --- | --- | --- |
| **Controls** | Mean 4985.70  Std D 4498.3 | 6.55  1.12 | 8.76  2.17 | 413.21  569.72 |
| **Family Unaffected Heterozygotes** | Mean 7384.68  SD 5465.31  ES 0.48 | 7.5  2.3  0.55 | 7.5  2.08  0.59 | 854.94  455.81  0.86 |
| **Family Unaffected Homozygotes** | Mean 6272.26  SD 3841.71  ES 0.30 | 7.96  1.4  0.36 | 6.57  1.13  1.32 | 915.85  724.2  0.77 |
| **Patients** | Mean 4283.33  SD 4561.88  ES 0.155 | 8.58  4.62  0.70 | 4.00  1.82  2.39 | 1762.25  1575.70  1.26 |

SD = Standard Deviation ES = Effect Size
