# Supplementary material for: In memory of Professor Iain Wilkinson: cognitive and neuroimaging endophenotypes in a consanguineous schizophrenia multiplex family
Source: Psychol Med. 2022 Feb 7;53(7):3178–86. doi: 10.1017/S0033291721005250 (PMC10235651; doi:10.1017/S0033291721005250)
Supplement: Supplementary file 1 [file S0033291721005250sup.zip › S0033291721005250sup004.docx]

**Table 4** **Reaction Time –** Patients’ reactions were slower and they made more errors than healthy controls and unaffected heterozygotes and homozygotes.

|  | **% Correct Trials** | **Mean Correct Latency** |
| --- | --- | --- |
| **Controls** | Mean 99.30  SD 0.87 | 343.16  96.82 |
| **Family unaffected heterozygotes** | Mean 99.75  SD 0.5  ES 2.27 | 357.28  85.26  0.15 |
| **Family unaffected homozygotes** | Mean 98.57  SD 1.27  ES 0.69 | 337.41  64.09  0.07 |
| **Patients** | Mean 85.75  SD 18.73  ES 1.38 | 878.32  221.66  3.36 |

SD = Standard Deviation ES = Effect Size
